# An Evaluation of the Source and Content of Dupuytren’s Disease Information Available on the Internet

**DOI:** 10.7759/cureus.19356

**Published:** 2021-11-08

**Authors:** Kyle Plusch, Jack Carfagno, Daniel Givner, Daniel Fletcher, Daren Aita, Greg G Gallant, Jack Abboudi, Pedro Beredjiklian

**Affiliations:** 1 Hand Surgery, Rothman Orthopaedic Institute, Philadelphia, USA; 2 Orthopedic Surgery, Philadelphia College of Osteopathic Medicine, Philadelphia, USA; 3 Orthopedic Surgery, Sidney Kimmel Medical College, Philadelphia, USA

**Keywords:** content, quality, information, internet, dupuytren's disease

## Abstract

Introduction

The internet continues to expand in both size and number of users, and patients are using the internet with increasing frequency to research orthopedic conditions and treatment options. Despite the prevalence of patients searching for medical information, the quality of the available information varies substantially. The purpose of this study was to investigate the reliability and accuracy of the information available on the internet for Dupuytren’s disease. We hypothesized that the informational content found on the internet regarding this condition would be of acceptable quality.

Methods

The search phrasing “‘Dupuytren’ OR ‘Dupuytren’s’” was used to mimic how patients would likely search for information on the disease. These terms were entered into the five English-language search engines with the most frequent use on the internet. On each search engine, the first 50 URLs were recorded, including sponsored sites. The 250 total sites were filtered to remove duplicate sites and URLs linking to other search engines, resulting in a final list of 84 websites for informational scoring. A previously published information evaluation protocol was used to grade each website. Each site was graded according to these guidelines by two authors and scored based on authorship, content, disease summary, treatment options, pathogenesis, complications, and results. A third author resolved any conflict on authorship or content before analysis. The resultant “informational value” is the sum of the disease summary, treatment options, pathogenesis, complications, and results and can range from 0-100.

Results

The mean total information score for all sites was 47.5 out of 100 points. Forty-three (51.2%) of the websites evaluated were authored by a physician or academic institution, and thirty-four (40.5%) of the sites were commercial in nature. The final seven websites (8.3%) had nonphysician, unidentified, or lay authorship. Physician and academic institution authored websites had an average informational score of 55.5 out of 100 points, compared to 39.7 out of 100 for all other websites. This difference was statistically significant (p <0.01). The mean informational score for the 10 sponsored websites was 16.4 out of 100.

Conclusion

We concluded that internet information on Dupuytren’s disease is of poor quality and incomplete. Academic and physician authored sites have higher quality than commercial sites, but significant room for improvement still exists. Patients should be advised to identify the authorship of the websites they obtain information from and avoid advertisements and commercial sites.

## Introduction

Flexion contractures of the fingers characterize Dupuytren’s disease due to the progressive formation of fibrous nodules and cords in the palmar fascia [1]. The condition is named after the French surgeon Baron Guillaume Dupuytren, who described and operated on the condition in the 1830s. He described the proliferation of palmar fascia and aponeurosis, dispelling the previous idea of flexor tendon involvement [2,3]. Although it most commonly involves the fourth and fifth digits, this disorder can affect the palm and any finger in variable patterns. Patients present with difficulty performing daily tasks involving the hands, such as putting on gloves or washing their face [4]. Our understanding of the etiology and management of Dupuytren’s disease has improved since the condition was initially described, with new surgical and therapeutic treatments continuing to emerge [5,6]. Despite extensive research into the genetic and geographic factors that influence the disease, the pathophysiology remains largely unknown [7]. Like many other medical conditions, patients more frequently are seeking information to gain an understanding of their conditions through internet searches.

Since its inception in 1969, the internet has grown vastly in its size and number of users [8]. Pew Research Center identified that 52% of United States citizens had access to the internet in the year 2000, with this number increasing to 93% in the year 2021 [9]. Patients are using the internet with increasing frequency to research orthopedic conditions and treatment options. Burrus et al. reported that 84.9% of their orthopedic clinic patients had access to the internet, while 64.7% of those with access used the internet to obtain orthopedic information [10]. Despite the prevalence of patients using the internet to research their orthopedic conditions, the quality of the available information varies. The purpose of this study was to investigate the reliability and accuracy of the information available on the internet for Dupuytren’s disease. We hypothesized that the informational content found on the internet regarding this condition would be of acceptable quality.

## Materials and methods

The search phrase "'Dupuytren' OR 'Dupuytren's'" was used to mimic how patients would likely search for information on the disease. These terms were entered into the five most frequently used English-language search engines on the Internet: (Google, Yahoo, Bing, AOL, and Ask) [11]. On each search engine, the first 50 URLs were recorded, including sponsored sites. The 250 total sites were filtered to remove duplicates and URLs linking to other search engines, resulting in a final list of 84 websites for informational scoring. A previously published information evaluation protocol was used to grade each website [12].

Each site was graded according to this protocol by two authors and scored based on authorship, content, disease summary, treatment options, pathogenesis, complications, and results. A third author resolved any conflict on authorship or content before analysis. The resultant "informational value" is the sum of the disease summary, treatment options, pathogenesis, complications, and results and can range from 0-100.

Authorship

The primary author of the website information was evaluated and grouped into one of the following seven categories: (1) "academic" indicated a clearly stated affiliation with a university or research organization; (2) "physician" indicated the author or authors were physicians in an individual or group practice with no stated affiliation with any university or research organization; (3) "nonphysician" care providers included physical or occupational therapists, acupuncturists, chiropractors, and any other alternative medical providers; (4) "commercial site" signified a commercial website without a stated interest or advertisement for a specific product (typically, these websites are designed to provide medical information to the lay public); (5) "commercial product"identifies a website that was primarily designed to market a commercial product to treat or evaluate Dupuytren's disease; (6)** **"lay" indicated an identifiable author that did not belong to any of the prior categories and presented a non-commercial website to provide information about Dupuytren's disease; or (7) "unidentified"designated that the author was not listed on the website.

Content

The content regarding information, evaluation, treatment, and pathogenesis of Dupuytren's disease on each website was categorized into one of four groups: (1) "conventional" indicated that the site provided only information consistent with current knowledge of the condition as described in orthopedic literature; (2) "unconventional" denoting the site gave alternative, unverified information regarding the condition in addition to conventional knowledge without the intent for secondary commercial gains; (3) "misleading" indicated the site provided unconventional information along with the intent for secondary commercial gains; or (4) "noninformational" specifying that the site contained no patient-related information.

Informational value

Disease Summary (maximum, 30 points)

Three points each were received when any of the following ten items were mentioned or clearly described: weakness, stiffness, contracture, anatomy of the hand, palmar nodules, knuckle pads, decreased motion on physical examination, decreased strength on physical examination, normal progression of the disease, and diagnosis using x-ray and/or MRI.

Treatment Options (maximum, 20 points)

Five points each were received when any of the following treatment options were mentioned: splinting, physical therapy, oral anti-inflammatory medications, collagenase injections, surgery.

Pathogenesis (maximum, 20 points)

Five points were received for each of the following potential etiologies of the condition were mentioned: family history, ethnic background, overuse, idiopathic.

Complications of Treatment (maximum, 15 points)

7.5 points were received for each of the following categories mentioned: complications of operative treatment (such as progression of disease, stiffness, infection, or nerve injury), and complications of nonoperative treatment (such as progression of the disease, side effects of oral anti-inflammatory medication). Any mention of a complication in either category was sufficient to earn the full 7.5 points per treatment type.

Results of Treatment (maximum, 15 points)

7.5 points were received when the results of operative treatment were given, and 7.5 points were received when the results of nonoperative treatment were given. Again, any mention of results in either category was sufficient to earn the full 7.5 points per treatment type.

Statistical analysis was performed using the Student's t-test for continuous variables. Interobserver reliability was calculated by comparing agreement rates between authorship/content type and overall informational scores. Each site's total informational score used for analysis was the average of each site's two authors' grades.

## Results

Analyzing the 84 websites for authorship, 43 (51.2%) were authored by an academic institution or physician, and a nonphysician care provider authored only 1. 25 (29.8%) were part of a commercial site, 9 (10.7%) were solely advertising a commercial product, and 6 (7.1%) had lay authorship or were unidentified. When evaluating website content, 65 (77.4%) of all sites provided conventional information. 7 (8.3%) provided unconventional information, and 12 (14.3%) were misleading or noninformational. Notably, 43 out of 45 academically affiliated or physician authored sites offered conventional information; 18 out of 25 commercial sites offered conventional information, while eight out of nine sites promoting commercial products were either misleading or noninformational. A detailed breakdown of content and authorship is shown in Table 1.

**Table 1:** Authorship and content of websites

Content	Authorship							
	Academic	Physician	Nonphysician	Commercial site	Commercial product	Lay	Unidentified	Total
All sites	35	8	1	25	9	3	3	84
Conventional	34	7	0	18	1	3	2	65
Unconventional	0	1	1	5	0	0	0	7
Misleading	0	0	0	1	7	0	0	8
Noninformational	1	0	0	1	1	0	1	4

The mean total informational score for all 84 unique websites was 47.5 out of a possible 100. The mean scores for each specific content section are shown in Table 2, further broken down by authorship type. The mean informational score of academic and physician authored sites was 55.5, compared to 39.7 for the remaining sites with this difference being statistically significant (p<0.01). Academic and physician authored sites also led each scoring category. The 84 unique sites included 10 sponsored websites (typically appear with “ad” next to them on the search engine). Excluding the sponsored sites, the mean total informational score for the remainder of the sites was 51.7, and the mean score for the sponsored sites was 16.4. 

**Table 2:** Mean informational scores of websites

	Disease summary (max 30)	Treatment options (max 20)	Pathogenesis (max 20)	Complications of treatment (max 15)	Results of treatment (max 15)	Total
All sites	13.7	10.4	9.5	6.3	7.6	47.5
All sites excluding sponsored sites	14.3	11.4	10.4	7	8.6	51.7
Academic/physician authored only	14.4	12.8	10.9	7.8	9.6	55
Non-academic/physician authored	12.9	8.3	8.1	4.8	5.6	39.7
Sponsored sites	8.9	2.8	3.3	1.1	0.4	16.4

Regarding interobserver reliability, the mean overall average score differed by 2.4% between the two observers, indicating a high degree of reliability. 15 sites (18%) had authorship conflicts and 10 (12%) had content conflicts, which a third author resolved before analysis.

## Discussion

Our review of the top 50 search results for Dupuytren’s on the five most commonly used search engines resulted in 84 unique websites for grading. With an average total informational score of 47.5 out of 100, we conclude that internet information on Dupuytren’s disease is of poor quality and incomplete. Including all websites, only two of our categories for informational content exceeded a 50% average score (treatment options = 52%; results of treatment = 51%). Among the different authorship categories, academic and physician authored sites scored the highest in every category of informational content, but even these higher scoring sites only reached 64% in their highest-scoring categories (once again, treatment options and results of treatment). A similar study using a different scoring system from Zuk et al. in 2017 also found substantial shortcomings in the web-based patient information on Dupuytren’s disease [13]. Despite a different scoring system, their median score was nearly identical to ours, at 44% of the maximum. Zuk et al. also compared informational score with the website’s age and found no improvement in score over time. In 2015, Kelly et al. looked at multiple common hand pathologies, including Dupuytren’s, and found an average score of 55% for Dupuytren’s content on the internet, although only based on the top 25 websites [14].

The poor quality score that we found (48% of maximum score) is consistent with other orthopedic internet information studies, including rotator cuff tears (49% and 55%) [15,16], cervical disc herniation (39%) [17], scoliosis (51%) [18], carpal tunnel syndrome (53.8%) [19], and Kienbock disease (44% and 45%) [20,21]. Studies on rarer conditions appear to result in slightly higher scores; for example, Nassiri et al. found an average score of 66% for the top 45 websites pertaining to the pediatric orthopedic condition Legg-Calves-Perthes disease [22], and Winship et al. recorded a score of 76% for websites discussing osteochondroma [23]. These higher scores may be due to the lack of commercial sites related to these topics, leaving a higher percentage of academic sites with less commercial and advertising influence. The studies mentioned above use a variety of different scoring systems to assess the quality of information. A commonly used scale is the DISCERN grading system [24], consisting of 16 questions graded on a five-point Likert scale for a maximum score of 80. Unlike our system, the scale is not tailored to any one topic, and its questions are based on general qualities about the site instead of specific informational criteria.

In addition to the quality of information, the readability of websites is another important consideration. Many studies have assessed readability based on the reading level of the information. A commonly used scale is the Flesch-Kincaid scale [25], with the recommended reading level for patient information from sixth to eighth grade [26]. Santos et al. analyzed the reading level of the top 10 websites for Dupuytren’s information in 2017 and found an average reading grade level of 10.2, with none of the websites falling into the recommended reading level [27]. This is similar to the findings of various other studies that reviewed the reading level of orthopedic information on the internet [28-31]. Considering the overall low-quality scores combined with an elevated reading level, patients are unfortunately left with websites that are difficult to comprehend while not providing complete information. 

Of the initial 250 sites from the five search engines, 111 were sponsored advertisements. Many informational studies exclude these sites, but their prevalence across all five of the search engines and our desire to provide a complete assessment of the websites that a patient may encounter led us to include these in our analysis. The vast majority of these ads were repeated across search engines, and many linked to a different search engine rather than an informational website. After excluding these sites, only 10 remained for scoring. Of these 10 sites, six advertised a commercial site or commercial product, and three linked to an academic group focused on Dupuytren’s research. Five of the sponsored sites contained unconventional, misleading, or noninformational content, resulting in a low average informational score of 16.4. A study on the quality of carpal tunnel information from Lutsky et al. in 2013 also included sponsored websites and found a similar average informational score of 14.5 using the same scale as in this study [19].

There are several limitations to this study. Primarily, the grading scale used provides an objective measure of completeness but has no objective measure of accuracy. A website that mentions all of the possible grading points on our scale while also mentioning other extraneous incorrect statements would receive a higher score than a website that mentioned fewer points but with 100% accuracy. Despite the lack of a separate accuracy measurement, the fact that specific criteria used in each grading category that were objectively factual regarding Dupuytren’s disease ensured that accurate statements were made on the websites to score points. Secondly, we did not exclude peer-reviewed articles or websites that required a subscription to access the full text (such as a Medscape article or an article on PubMed where only the abstract is visible for free). While many other internet quality studies exclude these, we wanted to have a complete picture of all sites that a patient may encounter. We did not want to assume that patients would have paid access to these sites, so these articles likely scored far lower than their full-text versions would have scored. Finally, we did not follow any links on the websites to find more information. There is a wide variety in website architecture, with some sites having additional information buried within multiple pages on one overall website. In order to not assume how thoroughly an average patient would search through a website, we only scored what was immediately visible on each link. 

## Conclusions

The internet will inevitably continue to expand as a source of medical information, but little evidence suggests that this information’s quality will substantially improve. While individual physicians cannot be held responsible for the quality of internet information, they can guide their patients to help them avoid misleading information and noninformational advertisements. Strategies to ensure patients only receive quality information can include providing office-made informational packets that provide complete information regarding the disease and promoting academic and physician authored websites. While any one individual site may not provide sufficient information, referencing a combination of only academic or physician-authored sites will provide a more accurate and complete picture for a patient. There remains substantial room for improvement in these sites, and the private practices and academic groups that provide informational content should strive to monitor and improve upon the content they publish.
